# “From Sensation to Mental Health: The PROFOCO-TEA as an Auxiliary Tool in the Psychiatric Diagnosis of Autism.”

**DOI:** 10.1192/j.eurpsy.2026.10811

**Published:** 2026-07-31

**Authors:** M. V. Stilpen, D. Cardilli, D. R. Molini-Avejonas

**Affiliations:** Rehabilitation Sciences., USP, São Paulo, Brazil

## Abstract

**Introduction:**

Autism Spectrum Disorder (ASD) includes underestimated **sensory comorbidities**. Children with ASD may have hypo- or hypersensitivity, which impacts their interaction with the environment. This study, therefore, investigates the prevalence of these **comorbidities** using the Cognitive Speech-Language-Hearing Protocol (PROFOCO-TEA) to map needs and improve clinical intervention.

**Objectives:**

The objective of this study was to investigate sensory alterations in children with Autism Spectrum Disorder (ASD), using the Cognitive Speech-Language-Hearing Protocol for Autism (PROFOCO-TEA).

**Methods:**

The study’s methodology was designed to ensure data reliability. It included **45 caregivers** of children with Autism Spectrum Disorder (ASD), aged **2 to 12**, in Petrópolis, Rio de Janeiro. A **convenience sample** was used for selection, and caregivers with cognitive impairments were excluded. Data collection was conducted **in person** to ensure a standardized and consistent application of the protocol. The study adhered to all **ethical principles**, was approved by the USP Research Ethics Committee, and required voluntary participation with the signing of a **Free and Informed Consent Form**.

**Results:**

The study’s results revealed sensory challenges in children with Autism Spectrum Disorder (ASD), with the visual modality standing out significantly at 89.1%. Other modalities also presented high incidences, such as tactile (73.9%), gustatory (64.4%), vestibular (58.7%), proprioceptive (48.9%), auditory (43.5%), and olfactory (39.1%). The sum of these percentages exceeded the total number of participants, reflecting the complexity of sensory profiles in ASD, where it is common for a child to present difficulties in multiple modalities simultaneously. This overlap of sensory challenges is a fundamental characteristic of the spectrum and should be considered in the planning of any intervention.

**Conclusions:**

The Cognitive Speech-Language-Hearing Protocol for Autism Spectrum Disorder (PROFOCO-TEA) emerges as an innovative and essential resource. The tool stands out for its viability and effectiveness in identifying the predominant sensory aspects in children with ASD, which are frequently neglected. By thoroughly investigating sensory difficulties, the protocol offers a precise map of the child’s individual needs, enabling a more assertive and effective speech-language-hearing intervention. Ultimately, the PROFOCO-TEA transforms assessment into a process that not only identifies challenges but also guides the creation of personalized support strategies.

**Disclosure of Interest:**

None Declared

